# Paeonol Relieves Chronic Neuropathic Pain by Reducing Communication Between Schwann Cells and Macrophages in the Dorsal Root Ganglia After Injury

**DOI:** 10.3390/ijms26093964

**Published:** 2025-04-22

**Authors:** Xin Li, Zifeng Zhuang, Yuting Hao, Shaozi Lin, Junyan Gu, Shiquan Chang, Lin Lan, Guoping Zhao, Di Zhang

**Affiliations:** College of Traditional Chinese Medicine, Jinan University, Guangzhou 510632, China; snsmbnsr201@163.com (X.L.); zifengzhuang0709@163.com (Z.Z.); shaozilin2024@163.com (S.L.); gujunyan0226@163.com (J.G.); changsq1113@163.com (S.C.); linlan0630@163.com (L.L.)

**Keywords:** Paeonol, IL-34, CSF1R, CellChat, CCI, single-cell sequencing

## Abstract

This study investigated the mechanism underlying Paeonol’s therapeutic efficacy against neuropathic pain. GSE158892 dataset data were used to conduct a scRNA-seq analysis. In cell experiments, Schwann cells and macrophages were utilized to examine pain pathogenesis using specific inhibitors. Thirty-two SD rats were randomly divided into four groups: sham, chronic constriction injury (CCI), ibuprofen, and Paeonol. Behavioral tests combined with ELISA, PCR, western blot, immunohistochemistry, and immunofluorescence analyses were conducted. CellChat analysis demonstrated that, following peripheral nerve injury, Schwann cells secreted IL-34, which interacted with CSF1R on macrophages, leading to the infiltration and activation of macrophages. Paeonol reduced IL-34 production by Schwann cells induced with LPS. Conditioned medium from LPS-stimulated Schwann cells treated with Paeonol did not cause macrophage proliferation or migration, activation of the CSF1 pathway, or ROS production. In CCI rats, Paeonol alleviated mechanical and cold hyperalgesia, while reducing the production of serum inflammatory mediators. Additionally, Paeonol decreased the expression levels of IL-34, CSF1R, phosphorylated ERK (p-ERK), phosphorylated NF-κB (p-NF-κB), and components of the NLRP3 inflammasome in the dorsal root ganglia of CCI rats. Conclusion: Alleviation of neuropathic pain by Paeonol treatment may be achieved by inhibiting the IL-34–CSF1R interaction, suppressing Schwann cell–macrophage interactions, and reducing DRG neuroinflammation.

## 1. Introduction

Neuropathic pain (NP) remains a major challenge for healthcare professionals due to its heterogeneous etiology. Guidelines issued by the National Institute for Health and Clinical Excellence (NICE) recommend the utilization of nonsteroidal anti-inflammatory drugs (NSAIDs) for neuropathic pain relief [1]. However, resistance to NSAIDs and side effects including dizziness, drowsiness, allergic reactions, and hepatic or gastrointestinal dysfunction continuously impact patients [2,3]. Therefore, there is an urgent need for new drugs for the treatment of NP. Nerve injury or malfunction results in localized release of neurotransmitters, neurotrophic factors, cytokines, and chemokines. These substances enhance the excitability and sensitivity of primary sensory neurons by reducing the threshold for peripheral nociceptor activation, thereby causing peripheral sensitization [4,5].

Paeonol is present in the traditional Chinese medicine Paeonia lactiflora. Previous studies have shown that it has multiple biological activities, such as antioxidant, anti-aging, and anti-inflammatory effects [6], and can alleviate pain caused by CCI in rats [7,8]. However, the underlying mechanism by which Paeonol relieves neuropathic pain is not clear. In the pathogenesis of NP, dorsal root ganglia (DRG) neurons transmit pain information to the spinal cord, and the DRG is a key node for the transformation of peripheral sensitization into central sensitization [9]. The proliferation of macrophages in the DRG ipsilateral to the lesion is an important cause of peripheral sensitization [10]. Following peripheral nerve injury, Schwann cells in the DRG produce various chemokines and recruit macrophages to the DRG on the injured side [11,12]. Schwann cells promote inflammatory reactions by secreting CSF1, IL-34, and SCF in peripheral neuropathy [12]. Activated macrophages secrete some inflammatory factors, such as IL-1β, IL-6, IL-18, TNFα, CXCL1, and CCL2, that increase the sensitivity of sensory neurons [13], thereby causing and intensifying peripheral sensitization. Macrophage activation requires the interaction of the CSF-1 receptor with CSF-1 and IL-34 [14]. These interactions initiate a cascade of signaling pathways that facilitate macrophage differentiation, proliferation, survival, as well as maintenance of normal macrophage function [15,16,17].

In this study, bioinformatics analysis of Schwann cells and macrophages revealed IL-34/CSF-1R involvement in pain pathogenesis. Furthermore, our investigation into Schwann cell–macrophage crosstalk in the DRG demonstrated that Paeonol attenuates this interaction through modulation of the IL-34/CSF-1R axis. Finally, preclinical animal studies confirmed the compound’s analgesic effects on neuropathic pain.

## 2. Results

### 2.1. The Proportion of Macrophages in the DRG Is Increased After Peripheral Nerve Injury

**Figure 1:**
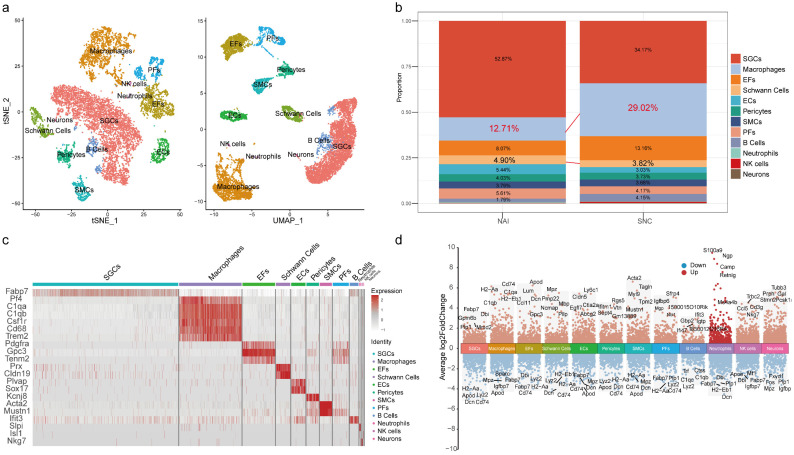
Classification of major cell types within the DRG. (**a**) Annotated UMAP and tSNE plots for DRG cells. (**b**) The proportions of various types of cells in the DRGs. (**c**) Heatmap of cell type markers. (**d**) Differentially expressed genes among cell types (hypervariable genes).

Cell annotation delineated 27 clusters. Based on known markers (Table 1 and Figure 1c) and differentially expressed genes (Figure 1d), these cells were categorized into 12 cell types (Figure 1a), described as macrophages (Pf4, C1qa, C1qb, Csf1r, Cd68, Trem2), smooth muscle cells (SMCs, Acta2, Mustn1), satellite glial cells (SGCs, Fabp7), NK cells (Nkg7), neutrophils (Slpi), epineurial fibroblasts (EFs, Pdgfra), perineurial fibroblasts (PFs, Gpc3, Tenm2), neurons (Isl1), endothelial cells (ECs, Plvap, Sox17), Schwann cells (Prx, Cldn19), B cells (Ifit3), and pericytes (Kcnj8). There were 789 macrophages (12.71%) and 1246 macrophages (29.02%) detected in the DRG after injury. In addition, there were 304 and 170 Schwann cells, accounting for 4.90% and 3.82% of all cells in the two groups, respectively (Figure 1b, and Table 2).

### 2.2. IL-34 Is Secreted by Schwann Cells and Taken Up by Macrophages in the DRG After Nerve Injury

**Figure 2:**
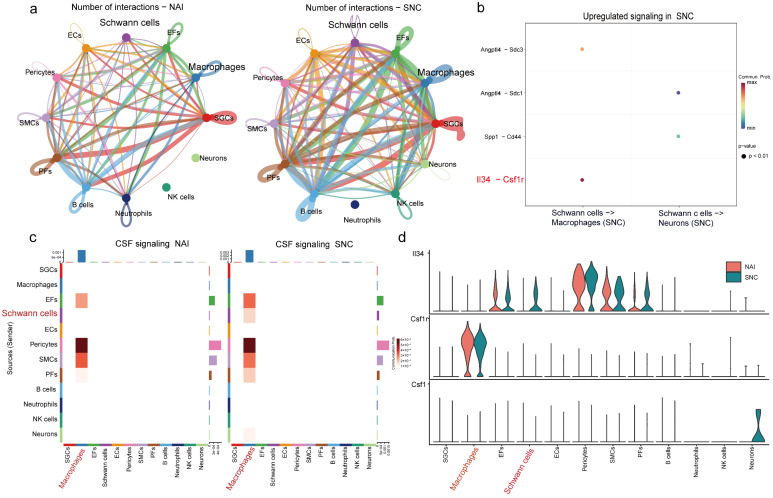
Communication among various cells in the DRG. (**a**) Number of cell–cell interactions. (**b**) Bubble plot of Schwann cell–macrophage ligand–receptor interactions. (**c**) Cell–cell interaction diagram for the CSF signaling pathway. (**d**) Violin plots of CSF1R, IL-34, and CSF1 levels in various types of cells.

CellChat was used to analyze interactions among cell types in the DRG (Figure 2a), showing increased cell–cell communication following nerve injury. Given that Schwann cells and macrophages play a vital role in peripheral sensitization [18], we focused on their interaction. The results revealed that IL-34 produced by Schwann cells targets macrophage CSF1R receptors (Figure 2b). Schwann cell–derived IL-34 activated CSF signaling in macrophages post-injury (Figure 2c). We examined the expression of CSF1R and its ligands (CSF1/IL-34) across cell types (Figure 2d), showing that CSF1R expression was macrophage-specific and correlated with elevated IL-34 levels after injury. CSF1, another CSF1R ligand, was expressed in neurons (Figure 2d).

### 2.3. The Impact of Paeonol on the Viability of RSC96 Cells

**Figure 3:**
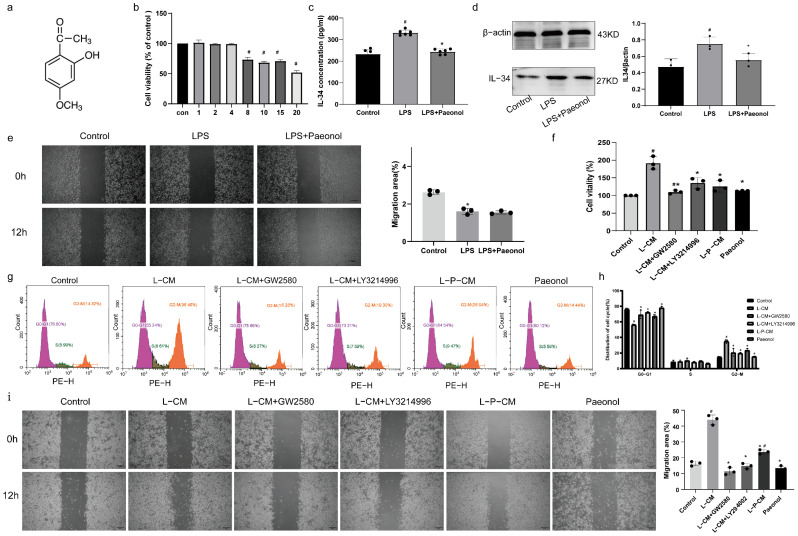
The level of IL-34 secreted by RSC96 cells affects macrophage proliferation and migration. (**a**) The chemical structure of Paeonol. (**b**) Effects of 1, 2, 4, 8, 10, 15, and 20 μm Paeonol on RSC96 cells. (**c**) IL-34 levels in the supernatant of RSC96 cells. (**d**) IL-34 expression in RSC96 cells. (**e**) LPS and Paeonol do not affect the migration ability of RAW264.7 cells. (**f**) Analysis of RAW264.7 cell proliferation. (**g**,**h**) Cell cycle analysis of RAW264.7 cells by PI staining. (**i**) Analysis of RAW264.7 cell migration by wound healing assay. Scale bar = 200 μm. #, compared with the sham group. *, compared with the CCI group. *p* < 0.05.

The chemical structure of Paeonol is depicted in Figure 3a. At concentrations of 1, 2, and 4 μM, Paeonol showed no significant impact on RSC96 cell viability. However, treatment with 8 μM Paeonol induced a marked reduction in cell viability (Figure 3b, *p* < 0.05). Based on these findings, 4 μM was selected as the working concentration for subsequent experiments.

### 2.4. Paeonol Inhibits the Secretion of IL-34 by RSC96 Schwann Cells Stimulated by LPS

The experimental results showed elevated IL-34 concentrations in RSC96 cell culture supernatants following lipopolysaccharide (LPS) stimulation across treatment groups. Paeonol treatment significantly reduced this elevation (Figure 3c, *p* < 0.05). Notably, LPS-stimulated Schwann cells exhibited markedly increased IL-34 protein levels (Figure 3d, *p* < 0.05), while combined LPS and Paeonol treatment resulted in significantly lower IL-34 expression (Figure 3d, *p* < 0.05).

### 2.5. LPS and Paeonol Do Not Affect the Migration Ability of RAW264.7 Cells

We evaluated the migratory capacity of RAW264.7 cells treated separately with LPS or Paeonol. The results revealed that neither LPS nor Paeonol had a pro-migratory effect on RAW264.7 cells (Figure 3e).

### 2.6. L-P-CM Failed to Trigger the Proliferation of RAW264.7 Cells

We assessed the effects of L-CM on RAW264.7 macrophages using CCK-8 assay and PI-based cell cycle analysis. The CCK-8 data showed significantly enhanced viability of L-CM–treated cells (Figure 3f, *p* < 0.05). Supplementation of L-CM–treated cultures with GW2580 (CSF1R inhibitor) and LY321499 (ERK inhibitor) markedly attenuated this proliferative effect (Figure 3f, *p* < 0.05). L-P-CM treatment resulted in no significant viability difference compared to controls (Figure 3f, *p* > 0.05). Cell cycle profiling revealed an increased G2-S phase proportion in the L-CM group (Figure 3g,h, *p* < 0.05), which was decreased by GW2580 and LY321499 supplementation (Figure 3g,h, *p* < 0.05). Notably, the L-P-CM group exhibited a significantly decreased G2-S phase proportion versus the L-CM group (Figure 3g,h, *p* < 0.05).

### 2.7. L-P-CM Does Not Notably Promote the Migration of RAW264.7 Cells

The wound healing assay demonstrated that L-CM–treated RAW264.7 macrophages displayed significantly higher migration rates compared to untreated controls (Figure 3i, *p* < 0.05). However, this migration rate was substantially weakened when comparing GW2580- or LY321499-supplemented L-CM groups to the L-CM only group (Figure 3i, *p* < 0.05). Although L-P-CM treatment enhanced macrophage migration relative to baseline controls, the achieved migration rate remained statistically lower than that observed in the standard L-CM treatment group (Figure 3i, *p* < 0.05).

### 2.8. Immunofluorescence Colocalization Analysis of CSF1R and IBA-1

**Figure 4:**
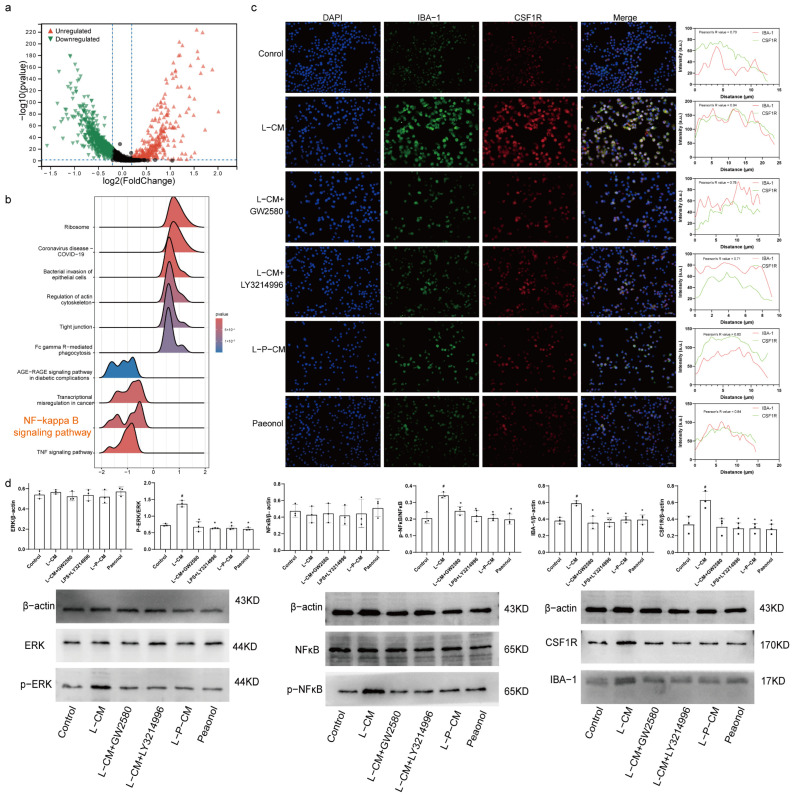
L-P-CM affected the CSF1R/ERK/NFKB pathway in macrophages. (**a**) Differential gene expression analysis of macrophages. (**b**) KEGG analysis. (**c**) Immunofluorescence colocalization analysis of IBA-1 and CSF1R. Scale bar = 200 μm. (**d**) Western blot analysis of CSF1R, IBA-1, ERK, p-ERK, NF-κB, and p-NF-κB expression. #, compared with the sham group. *, compared with the CCI group. *p* < 0.05.

KEGG analysis of differentially expressed genes in macrophages (Figure 4a) revealed injury-induced activation of the NF-κB signaling pathway in macrophages (Figure 4b). Immunofluorescence analysis showed elevated IBA-1 and CSF1R levels in L-CM–treated RAW264.7 cells. These increases were suppressed by GW2580, LY321499, and L-P-CM. Profile plots indicated that L-CM treatment increased CSF1R–IBA1 colocalization. The L-CM group showed higher Pearson correlation coefficients (Figure 4c). L-CM enhanced CSF1R-IBA1 colocalization, while GW2580, LY321499, and L-P-CM reversed this effect.

### 2.9. L-P-CM Fails to Activate the CSF1R/ERK/NF-κB Pathway in RAW264.7 Cells

The levels of CSF1R, ERK1(Mapk3), ERK2(Mapk1), NF-κB(Rela), and IBA-1(Aif1) increased after injury (Figure S1, *p* < 0.05). Compared to control groups, RAW264.7 cells treated with L-CM showed dramatically increased protein levels of CSF1R, p-ERK, p-NF-κB, and IBA-1 (Figure 4d, *p* < 0.05). GW2580 or LY321499 supplementation pf L-CM–treated cells significantly reduced CSF1R, p-ERK, p-NF-κB, and IBA-1 protein levels (Figure 4d, *p* < 0.05). The L-P-CM group also exhibited lower CSF1R, p-ERK, p-NF-κB, and IBA-1 levels than the L-CM group (Figure 4d, *p* < 0.05). However, ERK and NF-κB expression showed no intergroup differences (Figure 4d, *p* > 0.05).

### 2.10. L-P-CM Significantly Curbs Activation of the NLRP3 Inflammasome in RAW264.7 Cells

**Figure 5:**
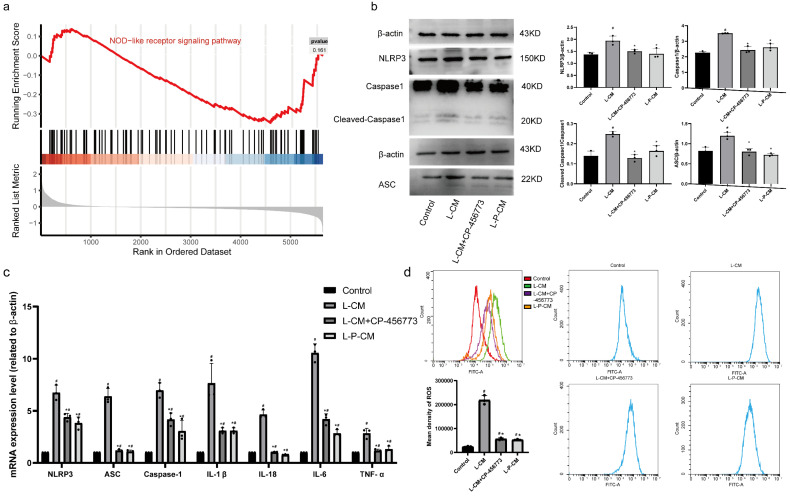
L-P-CM affected macrophages by inhibiting NLRP3. (**a**) GSEA analysis. (**b**) Western blot analysis of NLRP3 inflammasome components. (**c**) qPCR analysis of RAW264.7 cells. (**d**) Flow cytometry analysis. #, compared with the sham group. *, compared with the CCI group. *p* < 0.05.

GSEA analysis (Figure S2) revealed activation of the NOD-like receptor signaling pathway after injury (Figure 5a). The expression levels of caspase, NLRP3, IL-1β, IL-18, ASC, and caspase1 showed significant upregulation after injury (Figure S1, *p* < 0.05).

The levels of caspase1, ASC, and NLRP3 in L-CM–treated cells were higher than in controls (Figure 5b, *p* < 0.05); however, the addition of CP-45677 inhibited these changes (*p* < 0.05). L-P-CM treatment induced no significant increase in the above-mentioned proteins (Figure 5b, *p* > 0.05). PCR quantification revealed elevated mRNA levels of NLRP3, TNF-α, ASC, IL-18, IL6, caspase1, and IL-1β compared to control RAW264.7 cells (Figure 5c, *p* < 0.05), which were reversed by CP-45677. L-P-CM–treated RAW264.7 cells showed substantially decreased mRNA levels of these NLRP3 inflammasome components and cytokines (Figure 5c, *p* < 0.05).

### 2.11. L-P-CM Reduces ROS Production by RAW264.7 Macrophages Induced by NLRP3 Inflammasome Activation In Vitro

Flow cytometry analysis revealed significantly elevated ROS levels in L-CM–treated RAW264.7 macrophages (Figure 5d, *p* < 0.05). CP-45677 treatment significantly reduced ROS levels relative to the L-CM group (Figure 5d, *p* < 0.05). ROS levels in L-P-CM–treated cells were also markedly lower than in the L-CM group (Figure 5d, *p* < 0.05). However, neither L-P-CM nor L-CM treatment restored ROS levels to baseline control values.

### 2.12. Paeonol Relieves Mechanical Pain and Cold Hyperalgesia in CCI Rats

**Figure 6:**
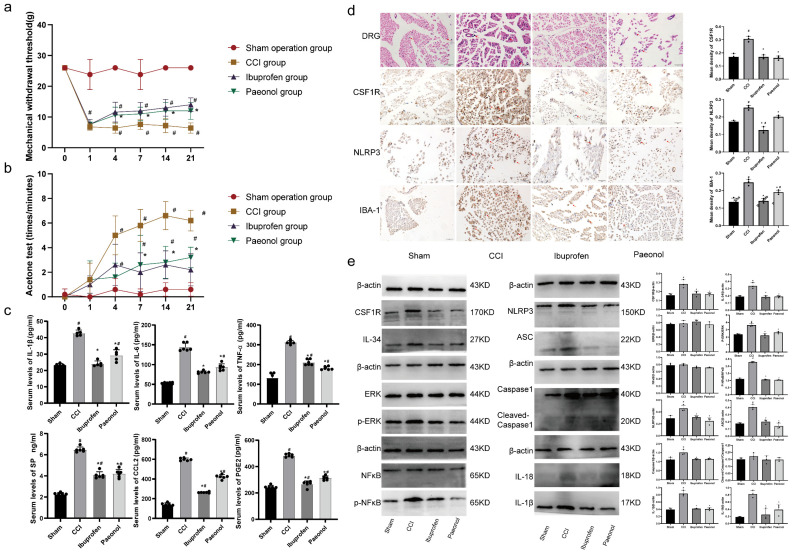
Paeonol treatment of chronic neuropathic pain. (**a**,**b**) Behavioral test. (**c**) Serum ELISA analysis. (**d**) H&E staining of DRG tissues and immunohistochemical staining for CSF1R, NLRP3, and IBA-1. The red arrows indicate monocytes/macrophages. (**e**) Western blot analysis of ERK, p-ERK, NF-κB, p-NF-κB, IL-1β, NLRP3, ASC, aaspase1, cleaved-caspase1, CSF1R, IL-34, and IL-18 expression. #, compared with the sham group. *, compared with the CCI group. *p* < 0.05.

On postoperative day 1, CCI rats exhibited significant mechanical hyperalgesia, with a markedly reduced paw withdrawal threshold (PWT) on the operated side (Figure 6a, *p* < 0.05). From days 4–21 post operation, CCI rats continued to exhibit lower PWTs compared to sham controls (Figure 6a, *p* < 0.05). Ibuprofen- and Paeonol-treated CCI rats showed higher PWT than untreated CCI counterparts during days 4–21 post surgery (Figure 6a, *p* < 0.05). However, neither treatment restored PWTs to normal levels.

In the acetone test, cold hyperalgesia showed no significant intergroup differences (Figure 6b, *p* < 0.05). CCI rats developed pronounced cold hyperalgesia by day 4 after surgery, with progressive worsening peaking at day 14, followed by improvement beginning at day 21 (Figure 6b, *p* < 0.05). From days 4 to 21 after surgery, ibuprofen- and Paeonol-treated CCI rats demonstrated reduced cold hyperalgesia compared to untreated CCI counterparts (Figure 6b, *p* < 0.05), though pain thresholds remained below baseline levels in both treatment groups.

### 2.13. Paeonol Reduces Serum Inflammatory Factor Levels in CCI Rats

ELISA analysis demonstrated significantly elevated serum levels of IL-6, PGE2, IL-1β, TNF-α, SP, and CCL2 in the CCI group compared to the sham group (Figure 6c, *p* < 0.05). Comparative analysis of CCI rat sera revealed notably decreased levels of IL-6, PGE2, IL-1β, TNF-α, SP, and CCL2 in ibuprofen- and Paeonol-treated groups compared to the CCI group (Figure 6c, *p* < 0.05).

### 2.14. Paeonol Reduces Levels of CSF1R, NLRP3, and IBA-1 in the DRG

H&E staining revealed sciatic nerve compression in all groups except the sham group. Liver and kidney H&E staining confirmed Paeonol’s safety, as intact tissue structures were observed across groups (Figure S3). DRG analysis showed increased macrophage infiltration in the CCI group versus the sham group (Figure 6d), and this effect was reduced in the ibuprofen- and Paeonol-treated groups. CCI significantly upregulated CSF1R, NLRP3, and IBA-1 expression (Figure 6d, *p* < 0.05), and Paeonol and ibuprofen effectively attenuated this effect (Figure 6d, *p* < 0.05).

### 2.15. Paeonol Curbs Activation of the CSF1R/ERK/NFκB Pathway and the NLRP3 Inflammasome

Western blot analysis of DRG levels of CSF1R, IL-34, ERK, p-ERK, NF-κB, p-NF-κB, NLRP3, ASC, IL-1β, caspase1, cleaved-caspase1, and IL-18 revealed elevated CSF1R, IL-34, p-ERK, p-NF-κB, caspase1, IL-1β, NLRP3, ASC, and IL-18 levels in CCI rats compared to the sham group (Figure 6e, *p* < 0.05). Paeonol- or ibuprofen-treated CCI rats showed significant reductions in these proteins (Figure 6e, *p* < 0.05). No intergroup differences were observed in ERK, NF-κB, or cleaved-caspase1 levels.

## 3. Discussion

This study presents compelling evidence indicating that Paeonol can alleviate neuropathic pain by mitigating the interaction between Schwann cells and macrophages. The key findings of this research are as follows. (1) Based on analysis of DRG cell communication, following peripheral nerve injury, Schwann cells secrete IL-34, which interacts with CSF1R receptors on macrophages. (2) Peripheral nerve injury causes an elevation in the mRNA expression of CSF1/ERK/NF-κB pathway components and NLRP3 inflammasome components in DRG macrophages. (3) Paeonol can decrease the quantity of IL-34 secreted by LPS-stimulated Schwann cells, inhibiting the subsequent interaction with CSF1R on macrophages. (4) Paeonol lessens macrophage infiltration and curbs activation of the CSF1 pathway and the NLRP3 inflammasome in CCI rats’ DRG.

Neuroinflammation plays crucial roles in the pathological mechanisms of NP. Activated macrophages release TNF-α, IL-1β, and IL-6. These cytokines act on neuronal receptors, boost peripheral neuronal excitability, and cause peripheral sensitization and hyperalgesia [19,20]. In diabetic peripheral neuropathy, higher levels of IL-1β, IL-6, and TNF-α promote oxidative stress, vascular dysfunction, and neuronal degeneration by activating important signaling pathways, including the NF-κB and MAPK pathways [21]. Activated TNF receptor 1 promotes p38MAPK expression, and knocking out p38αMAPK can relieve acute pain [22]. TNF receptor-associated factor 6 expression is increased in the spinal neurons and microglia of a CCI mouse model, and targeting the TRAF6/c-JUN/NF-κB pathway may be a prospective target for treating neuropathic pain [23]. Dahuang Fuzi Decoction relieves pain via inhibiting TNF-α and PI3K-AKT signaling [24]. Danggui Sini Decoction [25], ferulic acid [26], and ( +)-Catechin [27] suppress the NF-κB pathway, thereby relieving NP by reducing inflammation and promoting neural repair. Paeonol can inhibit the NF-κB pathway by downregulating epidermal growth factor receptor 2 [28]. In this study, Paeonol reduced the levels of inflammatory factors after CCI.

Crosstalk between Schwann cells and macrophages significantly impacts peripheral sensitization [29]. After peripheral nerve damage, Schwann cells release a variety of cytokines and chemokines to attract macrophages to the damaged area [30]. Activation of the IL34-CSF1R pathway is sufficient to induce macrophage migration [31]. In this study, analysis of scRNA data confirmed that DRG Schwann cells secrete IL-34 after nerve injury, and we verified its interaction with CSF1R on macrophages. Moreover, the mRNA expression levels of CSF1R, ERK1, ERK2, and NF-κB, components of the CSF1 signaling pathway, showed a trend toward upregulation in in macrophages after injury. Therefore, it is speculated that DRG Schwann cells trigger macrophage activation by promoting the interaction between IL34 and CSF1R. CCI increased the levels of IL34, CSF1R, p-NF-κB, and p-ERK, which was consistent with the scRNA analysis. Additionally, our previous studies demonstrated that stigmasterol and (+)-Catechin alleviate neuropathic pain by reducing Schwann cell–macrophage interactions in the DRG through modulation of the IL-34/CSF1R axis [32,33]. In this study we focused on the IL-34/CSF1R/P38MAPK/NF-κB pathway. Further in vitro experiments showed that LPS greatly increased IL- 34 secretion, while Paeonol treatment effectively decreased it. Moreover, CCK-8 assay and cell cycle analysis revealed that the proliferation of RAW264.7 cells treated with L-CM was notably increased, while macrophages treated with L-P-CM did not show remarkable signs of proliferation. In addition, Paeonol did not promote macrophage proliferation and migration. Moreover, the wound healing assay revealed that RAW264.7 cells treated with L-CM exhibited stronger migration ability, while RAW264.7 cells treated with L-P-CM did not show a noteworthy increase in migration. These findings show that Paeonol inhibits LPS-induced IL-34 release from Schwann cells, which could promote macrophage proliferation and migration.

Macrophage activation and inflammatory factor release are tightly connected to NLRP3 inflammasome activation [34], which is regulated by NF-κB during transcription, while activation of ERK triggers posttranslational modifications [35]. L-CM treatment significantly raised the levels of NLRP3, ASC, caspase1, IL-1β, and IL-18 in RAW264.7 cells. L-P-CM treatment did not lead to a significant increase in protein expression in RAW264.7 cells. CSFR1 activation causes ROS production [36,37], which causes nerve damage and increases neuronal excitability [38]. L-CM–cultured macrophages had much higher ROS levels than the control group, and NLRP3 inhibitors reversed this change. Treatment with GW2580, a CSF1R antagonist, can decrease ROS production by microglial cells [37]. Macrophages cultured in L-P-CM had lower ROS levels than those in the L-CM group due to reduced levels of IL-34. These results show that Paeonol may inhibit NLRP3 inflammasome activation and ROS production in macrophages by curbing LPS-induced IL-34 release from Schwann cells.

In vitro experiments showed that Paeonol could inhibit IL-34 secretion from RSC96 Schwann cells and block RAW264.7 cell proliferation and activation. Then, further in vivo verification studies were carried out in CCI rats. Behavioral tests showed that Paeonol eased mechanical and cold pain in CCI rats. ELISA analysis showed that it lowered the expression of inflammatory factors (IL-1β, IL-6, TNF-α, CCL2) and pain-related cytokines (SP, PGE2). This shows that Paeonol exerts analgesic effects by inhibiting inflammation, which is consistent with other studies [7,8]. DRG immunohistochemical staining and Western blot showed that Paeonol decreased the expression of IL-34/CSF1R signaling pathway components and NLRP3 inflammasome components in CCI rats. The above results suggest that alleviation of neuropathic pain by Paeonol may be achieved by inhibiting the IL-34–CSF1R interaction, suppressing Schwann cell–macrophage interactions, and reducing DRG neuroinflammation.

This study had some shortcomings. (1) Rat experiments were used to validate the pharmacodynamics, but only one dose was used. (2) Single-cell sequencing analysis suggested that IL-34/CSF1R plays a vital role in post–nerve injury pain. While Paeonol reduced IL-34 levels after LPS stimulation, we did not knock down IL-34, so we cannot rule out potential confounding effects from RSC96 cell secretions.

## 4. Materials and Methods

### 4.1. Reagents

LPS (L2880) was acquired from Sigma–Aldrich (Shanghai) Trading Co., Ltd. (Shanghai, China). LY3214996 (CSF1R and ERK inhibitor), GW2580 (CSF1R inhibitor), and CP-456773 (NLRP3 inhibitor) were acquired from Shanghai Yuanye Biotechnology Co., Ltd. (Shanghai, China). Paeonol (product code: H111081, with a purity of 99%) was procured from Shanghai Aladdin Biotechnology Co., Ltd. (Shanghai, China). Ibuprofen (H20066) was acquired from Jinan University First Affiliated Hospital. The Evo M-MLV RT Mix Kit (AG11012) and SYBR Green Premix qPCR Kit (AG11701) were acquired from Hunan Accurate Biotechnology Co., Ltd. (Changsha, China). JiangSu MEIMIAN Industrial Co., Ltd. (Yancheng, China) provided the IL-10 (MM-0647R1), PGE2 (MM-0068R1), TNF-α (MM-0180R1), SP (MM-0444R1), IL-1β (MM-0047R1), and IL-6 (MM-0190R1) ELISA kits. Antibodies were sourced as follows: β-actin (380624), caspase-1 (381016), and ASC (340097) from ZenBio (Durham, NC, USA); NLRP3 (WL02635) and IL-1β (WL02257) from BioDog (Porirua, New Zealand); IL-34 (31957) and CSF1R (38540) from Signalway Antibody (Greenbelt, MD, USA); CSF1R (GB11581) and IBA-1 (GB12105) from Servicebio (Wuhan, China); and ERK (A5029) and p-ERK (A5036) from Bimake (Houston, TX, USA).

### 4.2. Single-Cell Data Quality Control and Annotation

Two naïve mice’s L4 DRG (uninjured mouse) data and an SNC mouse’s L4 DRG (mouse with sciatic nerve injury) data were obtained from the GSE158892 dataset. First, R (v.4.3) and Seurat (v.4.4) were used to perform quality control with cells having >200 unique molecular identifiers (UMIs), <4000 genes, and <15% mitochondrial genes. Thirty principal components and a resolution of 0.8 achieved dimensionality reduction and clustering, producing 27 clusters. Subsequently, after detecting highly variable genes, identifying clusters, and generating UMAP dimplots, we conducted cell annotations via cell markers (Table 1) and Cell Marker 2.0 (http://bio-bigdata.hrbmu.edu.cn/CellMarker/, accessed on 15 March 2025) and Enrichr (https://maayanlab.cloud/Enrichr/, accessed on 15 March 2025).

### 4.3. Differential Gene Analysis of Macrophages

We conducted differential gene analysis of macrophage via “FindMarkers” (logFC.threshold = 0.2). A volcanic map displayed differentially expressed genes (logFC.threshold = 0.2, *p* < 0.05). These genes were subjected to Gene Set Enrichment Analysis (GSEA) and Kyoto Encyclopedia of Genes and Genomes (KEGG) analyses.

### 4.4. Analysis of Cell Communication in the DRG

Cell crosstalk in the DRG was analyzed using the CellChat R package (1.4.1). The interaction dataset within CellChatDB was designated as the reference database. Subsequently, communication probability was computed by means of a truncated mean of 20% (employing the function computeCommunProb, with type specified as “truncatedMean” and trim set to 0.2).

### 4.5. Cell Culture

RAW264.7 cells and RSC96 cells were cultured in DMEM supplemented with 10% fetal bovine serum and 1% penicillin-streptomycin (100 U/mL) in an incubator maintained at 37 °C with 5% CO₂. The RSC96 and RAW264.7 cells underwent subculturing two to three times per week and were used in experiments at passages eight to ten.

### 4.6. Experimental Grouping of RSC96 Schwann Cells and Preparation of Conditioned Medium

RSC96 cells were assigned to three groups: the control group, the LPS group (treated with 10 μg/mL LPS), and the LPS + Paeonol group (treated with 10 μg/mL LPS and 4 μM Paeonol).

To collect LPS-stimulated Schwann cell-conditioned medium, 1.0 × 10^6^ Schwann cells were plated onto 100 mm culture dishes and allowed to adhere overnight. After the medium was removed, 5 mL of serum-free medium containing Paeonol was added to the culture dish. Following a 24 h treatment period, the supernatant was harvested. After centrifugation at 1000× *g* for 5 min, the mixture was filtered through a 0.22 µm filter. The supernatant of Schwann cells treated with LPS was considered LPS-stimulated Schwann cell medium (L-CM), while the supernatant of Schwann cells treated with LPS+Paeonol was considered LPS+Paeonol–treated Schwann cell medium (L-P-CM).

### 4.7. Experimental Grouping of RAW264.7 Macrophages

RAW264.7 cells were used in two experiments. For the first experiment, the cells were divided into: the sham group (untreated control), the LPS group (treated with 10 μg/mL LPS), and the Paeonol group (treated with 10 μg/mL LPS + 4 μM Paeonol).

For the second experiment, the cells were divided into seven distinct groups: the control group (exposed to unconditioned medium), the L-CM group (exposed to L-CM), the L-CM+GW2580 group (treated with L-CM and 5 μM GW2580, a CSF1R inhibitor), the L-CM+LY3214996 group (treated with L-CM and 10 μM LY3214996, an ERK inhibitor), the L-CM+CP-456773 group (treated with L-CM and 10 μM CP-456773, an NLRP3 inhibitor), the L-P-CM group (exposed to L-P-CM), and the Paeonol group (exposed to unconditioned medium supplemented with Paeonol).

### 4.8. IL-34 Secretion by RSC96 Cells

Secretion of IL-34 into the supernatant of RSC96 cells from the control group, LPS group, and LPS+Paeonol group was assessed using a Rat Interleukin 34 ELISA Kit.

### 4.9. Analysis of Cell Viability

CCK-8 assay was conducted to evaluate the viability of RSC96 cells following treatment with Paeonol. In accordance with the instructions of the CCK-8 kit, the effects of Paeonol at concentrations of 1 μM, 2 μM, 4 μM, 8 μM, 10 μM, 15 μM, and 20 μM on the viability of RSC96 cells were examined.

In addition, we evaluated the effect of L-CM on the proliferation of RAW264.7 cells via CCK-8. RAW264.7 cells were seeded into 96-well plates in complete medium at a concentration of 5 × 10 cells/well. Once adhered, the cells were rinsed with PBS and then incubated at 37 °C with the conditioned medium collected as previously described. After 24 h of treatment, the medium was refreshed, and CCK-8 reagent was added. After 1 h, the OD value was measured using an Epoth multifunctional microplate reader.

### 4.10. Cell Cycle Analysis via Flow Cytometry

RAW264.7 cells were plated in six-well culture dishes at a density of 1 × 10^6^ per well and cultured with the conditioned medium for 24 h. Then, a cell suspension was prepared.

After washing, the pellet was resuspended in 2 mL 70% ethanol that had been precooled to −20 °C, and the cells were fixed at 4 °C for 30 min. After fixation, the cells were washed once more with PBS. Subsequently, RNase A (20 μg/mL) was added, and the cells were incubated at 37 °C for 30 min to ensure complete degradation of intracellular RNA. Afterward, the cells were resuspended in 500 μL PBS and 25 µL PI, placed on ice, and incubated in the dark for 30 min before staining for flow cytometry.

### 4.11. Macrophage Migration Assay

The migration of macrophages was evaluated by wound healing assay. RAW264.7 cells were seeded into six-well plates. Once a 90% confluent monolayer had formed, the monolayer was scratched with a pipette tip. The wound area was observed with a light microscope (Carl Zeiss AG, Oberkochen, Germany) at 0 and 12 h after scratching. Cell motility was computed using ImageJ (1.40).

### 4.12. Immunofluorescence

After culturing and treating with the conditioned medium, RAW264.7 cells were fixed with 4% paraformaldehyde and 0.5% Triton X-100. Subsequently, the cells were blocked for 1 h with 10% donkey serum PBS. Thereafter, they were incubated for 12 h at 4 °C in a humidified chamber with rabbit anti-CSF1R and mouse anti-IBA-1 primary antibodies at a dilution ratio of 1:200. After washing with PBS, the cells were incubated with double secondary antibody (1:400) for 50 min. Subsequently, the cells were rinsed with PBS, and DAPI staining solution was added. Following a 10 min incubation, all images were captured with an Olympus BX53 microscope.

### 4.13. Detection of ROS by Flow Cytometry

After culturing RAW264.7 cells in the conditioned medium, the cells were harvested, and a cell suspension was prepared. The cells were rinsed with PBS and then incubated with DCFH-DA diluted 1:1000 (10 μmol/L) in a 37 °C cell culture incubator for 20 min. Thereafter, the cells were rinsed three times and then subjected to flow cytometry analysis.

### 4.14. Subjects

SD rats (180–230 g) were acquired from the experimental center of Beijing Huafukang Co., Ltd. (Beijing, China). The rats were housed five per cage in the Animal Experiment Center of the School of Medicine, Jinan University. All animal experiments were conducted in strict adherence with to the guidelines set forth by the National Academy of Sciences, the National Institutes of Health (NIH), and the Committee on the Care and Use of Laboratory Animals (Ethics Committee of the Institute of Zoology at Jinan University), and were approved by the ethics committee with approval document number IACUC-20221114-02.

### 4.15. Neuropathic Pain Model

We chose to use a CCI model in this study [25,39]. After intraperitoneal injection of pentobarbital sodium, the right sciatic nerve was exposed and ligated using a 4.0 suture (four ligatures approximately 1 mm apart). Rats in the sham group had their sciatic nerves exposed, but not ligated.

In total, 32 rats were divided into four groups. The sham operation group (sham) and CCI group (CCI) were gavaged with 0.9% normal saline (6 mL/kg). The ibuprofen group was gavaged with ibuprofen (31.5 mg/kg). The rats in Paeonol group were gavaged with Paeonol (100 mg/kg) [8]. Treatment continued for 21 days.

### 4.16. Behavioral Tests

Mechanical pain sensitivity was evaluated using the von Frey filament test. Alterations in the mechanical pain threshold on the operative side were measured 1 day prior to the operation and on the 1st, 4th, 7th, 14th, and 21st days post operation. Before the test, the rats were placed in plastic cages and allowed to move freely. When the rats were still, a von Frey filament with a bending force of 2–26 g was applied to the paw of each rat. Rapid withdrawal of the paw in response to a filament was recorded as a positive result. The experimenter applied each filament 10 times at 2 min intervals and recorded the paw withdrawal count.

We used the acetone test to measure cold hyperalgesia in the rats. Before the experiment, the acetone was stored in a refrigerator at 4 °C for 3 h, and the rats were placed in a cage with a wire mesh floor (0.5 cm × 0.5 cm). The rats were allowed to move freely in a quiet environment at room temperature. When the rats were still, 100 μL of cold acetone was applied to the surface of the right hind paw, and the number of times the rat raised its foot within 2 min and the duration of foot lifting were recorded. The test was repeated three times at 15 min intervals.

### 4.17. H&E Staining

The ligated sciatic nerve tissue and the right DRG tissue were fixed in 4% paraformaldehyde for 24 h, embedded in paraffin, and sectioned at a thickness of 3 μm. The sections were deparaffinized in xylene and rehydrated through a series of ethanol solutions with concentrations of 100%, 90%, 80%, and 70%. Subsequently, the sections were rinsed in PBS. After staining, all images were captured with an Olympus microscope.

### 4.18. Measurement of Inflammatory Factor Levels in Rat Serum by ELISA

First, 200 µL of the blood supernatant was diluted to 1000 µL. Then, according to the manufacturer’s instructions, we measured concentrations via optical density.

### 4.19. Immunohistochemistry

Right DRG tissues sections were placed in sodium citrate antigen retrieval solution (1:1000 dilution; pH = 6), then blocked with BSA at 37 °C for 2 h and incubated with primary antibody at 4 °C for 12 h. After incubating with the corresponding secondary antibody, color development was performed by incubation with DAB for 5–10 min.

### 4.20. qRT–PCR Analysis of RAW264.7 Cells

RNA was isolated from cells using RNAiso Plus then reverse-transcribed to cDNA. The amplification conditions were as follows: an initial denaturation step at 95 °C for 1 min, followed by 40 cycles consisting of 10 s of denaturation at 95 °C and 30 s of annealing at 60 °C. The sequences of the primers employed are presented in Table 3.

### 4.21. Western Blotting

Right DRG tissues or RSC96 and RAW264.7 cells were homogenized in RIPA buffer, and the homogenate was centrifuged at 14,000× *g* for 15 min to extract total protein. Thirty micrograms of protein were separated by 10–12% SDS-PAGE and then transferred onto a PVDF membrane. The membrane was blocked with 5% milk for 1 h, incubated with the primary antibody at a dilution ratio of 1:1000 at 4 °C for 12 h, and then incubated with the secondary antibody at a dilution ratio of 1:10,000 for 1 h. Subsequently, the bands were visualized using a ChemiDoc XRS imager. Each experiment was conducted in triplicate.

### 4.22. Statistics

The results are presented as means with SEM from three independent experiments. Analysis of these data was performed using GraphPad Prism 9 and R version 4.3 software. The Kolmogorov–Smirnov test was used to evaluate the normality of the data. In cases where the data exhibited normal distribution and uniform variance, statistical comparisons among multiple groups were conducted using either one-way or two-way analysis of variance (ANOVA), followed by post hoc Bonferroni test for pairwise comparisons. A P value below 0.05 was deemed indicative of statistical significance.

## 5. Conclusions

Paeonol alleviates neuropathic pain, likely through modulating IL-34/CSF1R binding and suppressing Schwann cell–macrophage interactions, which reduces inflammatory mediator–driven peripheral sensitization in the DRG.

## Figures and Tables

**Table 1:** Cell Markers.

Cell Types	Markers
SGCs	Fabp7
Macrophages	Pf4, C1qa, C1qb, Csf1r, Cd68, Trem2
EFs	Pdgfra
Schwann Cells	Prx, Cldn19
ECs	Plvap, Sox17
Pericytes	Kcnj8
SMCs	Acta2, Mustn1
PFs	Gpc3, Tenm2
B Cells	Ifit3
Neutrophils	Slpi
NK cells	Nkg7
Neurons	Isl1

**Table 2:** The proportions and cell number of various types of cells in the DRG.

Cell Types	Naive	SNC
SGCs	52.87% (3282)	34.17% (1467)
Macrophages	12.71% (789)	29.02% (1246)
EFs	8.07% (501)	13.16% (565)
Schwann Cells	4.90% (304)	3.82% (164)
ECs	5.44% (338)	3.03% (130)
Pericytes	4.03% (250)	3.73% (160)
SMCs	3.79% (235)	3.68% (158)
PFs	5.61% (348)	4.17% (179)
B Cells	1.79% (111)	4.15% (178)
Neutrophils	0.53% (33)	0.21% (9)
NK cells	0.16% (10)	0.61% (26)
Neurons	0.11% (7)	0.26% (11)

**Table 3:** Primer sequences.

Gene	Forward Primer (5’->3’)	Reverse Primer (5’->3’)
β actin	CCTAGACTTCGAGCAAGAGA	GGAAGGAAGGCTGGAAGA
TNF-α	GCGTGTTCATCCGTTCTCTACC	TACTTCAGCGTCTCGTGTGTTTCT
Caspase1	TGAAAGACAAGCCCAAGGT	GAAGAGCAGAAAAGGAAAAA
NLRP3	CTGTCTCACATCTGCGTGTT	GTCTCCCAAGGCATTTTCT
IL-6	AGTTGCCTTCTTGGGACTGATGT	GGTCTGTTGTGGGTGGTATCCTC
IL1β	AGGAGAGACAAGCAACGACA	CTTTTCCATCTTCTTCTTTGGGTAT
IL-18	CTGGCTGTGACCCTATCTG	AAGCATCATCTTCCTTTTGG
ASC	AGACATCGGGAGGATTTTAC	GAGCACCACACTCAAGG

## Data Availability

The data used to support the findings of this study are available from the corresponding author upon request.

## Supplementary Materials

The following supporting information can be downloaded at: https://www.mdpi.com/article/10.3390/ijms26093964/s1.

## Abbreviations

Definition	Abbreviation
Apoptosis-associated speck-like protein containing a CARD	ASC
Angiopoietin-like 4	Angptl4
Chronic constriction injury	CCI
C-C motif chemokine ligand 2	CCL2
Colony-stimulating factor 1 receptor	CSF1R
Dorsal root ganglion	DRG
Extracellular signal-regulated kinase	ERK
Gene set enrichment analysis	GSEA
Interleukin-1	IL-1
Interleukin-1 beta	IL-1β
Interleukin-6	IL-6
Interleukin-18	IL-18
Interleukin-34	IL-34
Lipopolysaccharide	LPS
Kyoto Encyclopedia of Genes and Genomes	KEGG
Nuclear factor NF-kappa-B	NF-κB
NACHT, LRR, and PYD domain–containing protein 3	NLRP3
Reactive oxygen species	ROS
Tumor necrosis factor-α	TNF-α
