# Supplementary material for: Paeonol Relieves Chronic Neuropathic Pain by Reducing Communication Between Schwann Cells and Macrophages in the Dorsal Root Ganglia After Injury
Source: Int J Mol Sci. 2025 Apr 22;26(9):3964. doi: 10.3390/ijms26093964 (PMC12071476; doi:10.3390/ijms26093964)
Supplement: Supplementary file 1 [file ijms-26-03964-s001.zip › ijms-3540052-supplementary.pdf]

Supplementary Figures：

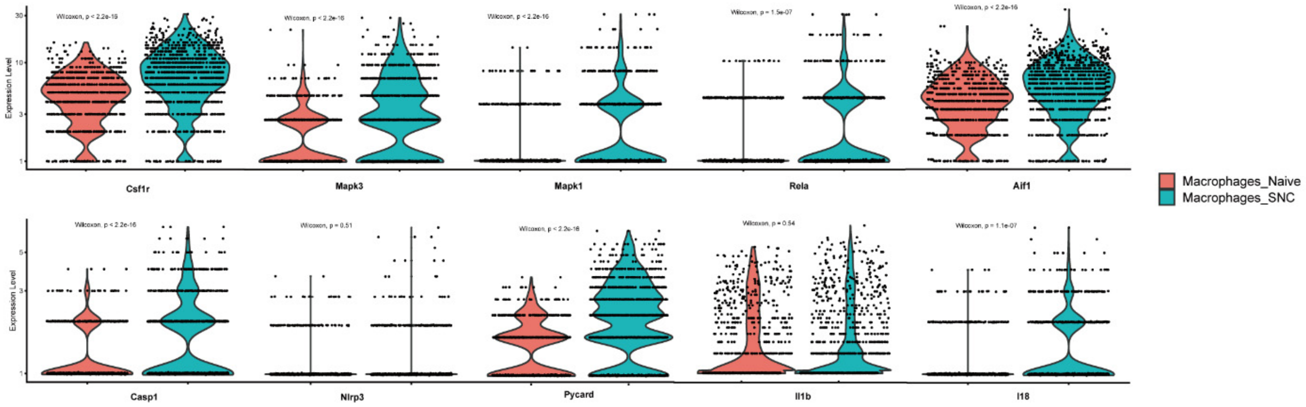

Figure S1. Violin plots showing the levels of CSF1R, ERK1(Mapk3), ERK2(Mapk1), NF-κB(Rela), IBA-1(Aif1), Caspase, NLRP3, IL-1β, IL-18, ASC, and Caspase1 following injury.

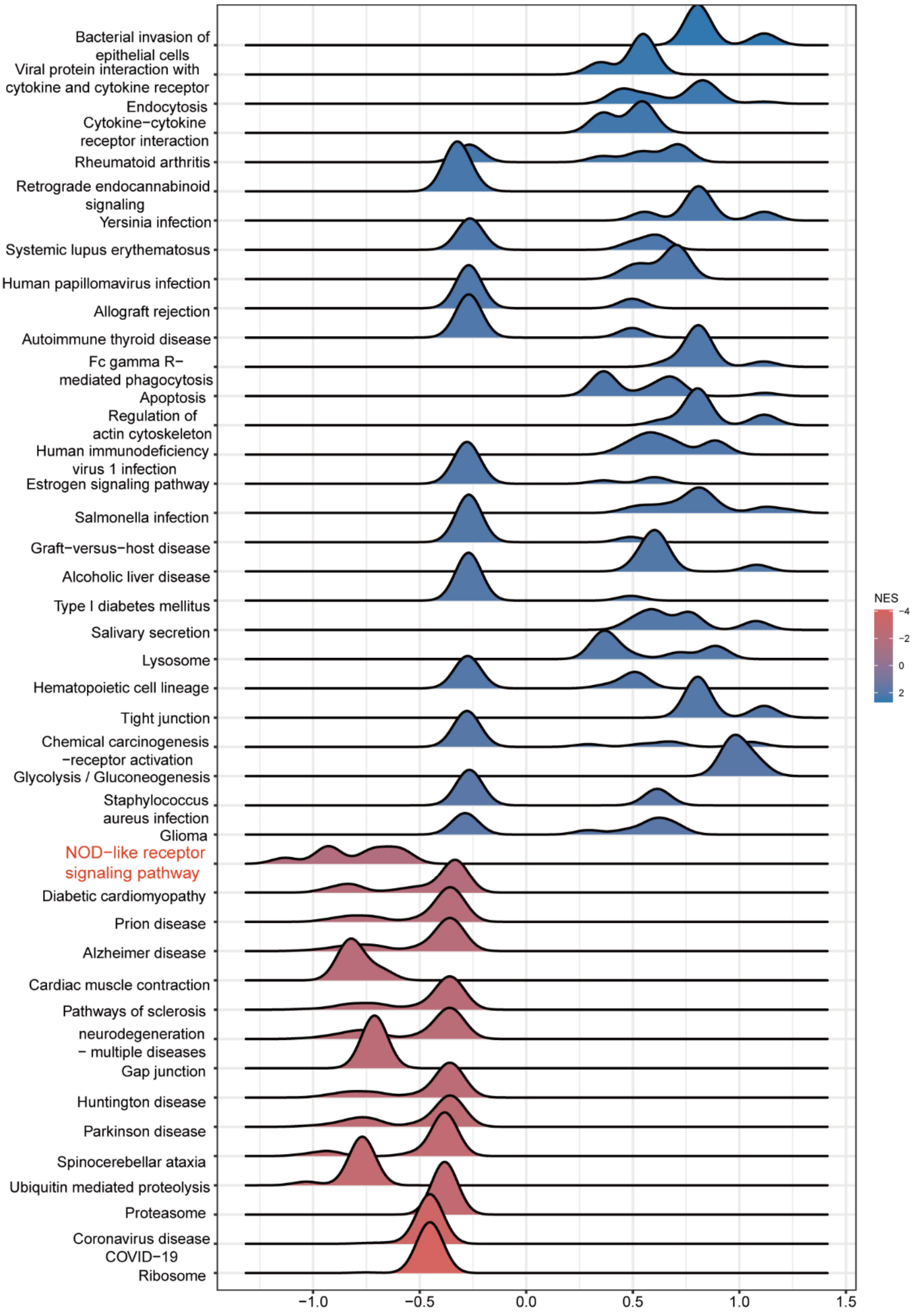

Figure S2. GSEA analysis revealed activated 'NOD-like receptor signaling pathway' after injuries

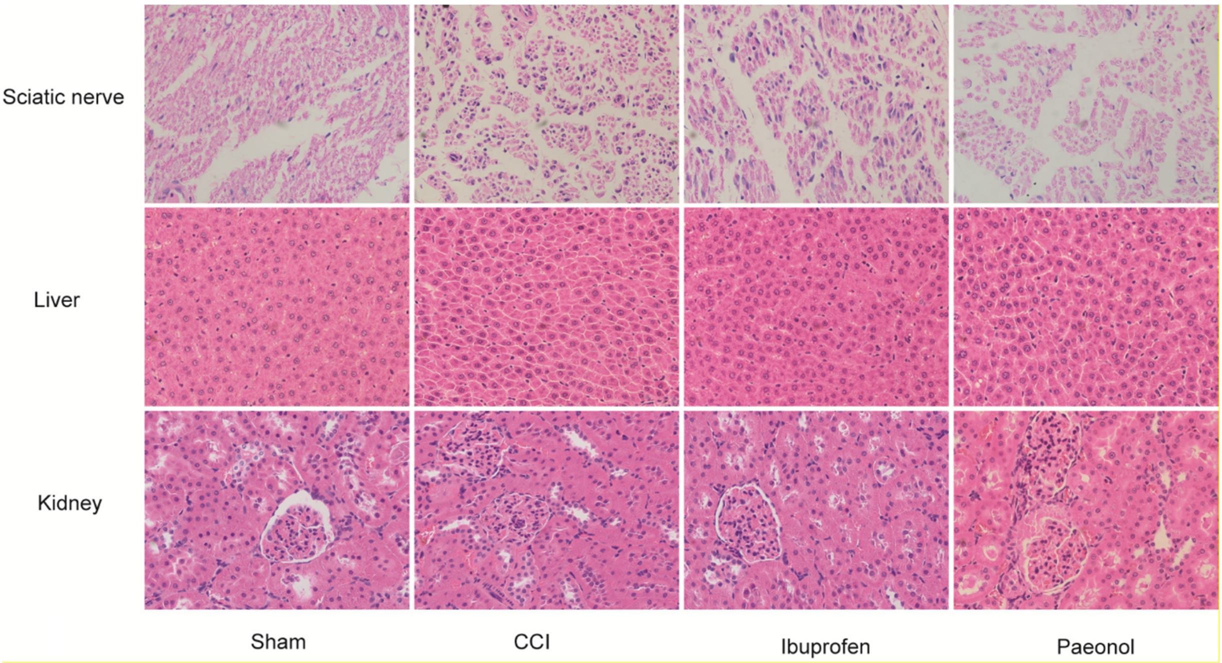

Figure S3. H&E staining of Sciatic nerve、liver and kidney.
